# The Contributions of Mu-Opioid Receptors on Glutamatergic and GABAergic Neurons to Analgesia Induced by Various Stress Intensities

**DOI:** 10.1523/ENEURO.0487-21.2022

**Published:** 2022-05-31

**Authors:** Yinan Du, Kexin Yu, Chuanting Yan, Chunling Wei, Qiaohua Zheng, Yanning Qiao, Yihui Liu, Jing Han, Wei Ren, Zhiqiang Liu

**Affiliations:** MOE Key Laboratory of Modern Teaching Technology, Shaanxi Normal University, Xi’an 710062, China

**Keywords:** forced swim stress, stress-induced analgesia, mu-opioid receptor, periaqueductal gray, β-endorphin

## Abstract

The endogenous opioid system plays a crucial role in stress-induced analgesia. Mu-opioid receptors (MORs), one of the major opioid receptors, are expressed widely in subpopulations of cells throughout the CNS. However, the potential roles of MORs expressed in glutamatergic (MOR_Glut_) and γ-aminobutyric acidergic (MOR_GABA_) neurons in stress-induced analgesia remain unclear. By examining tail-flick latencies to noxious radiant heat of male mice, here we investigated the contributions of MOR_GABA_ and MOR_Glut_ to behavioral analgesia and activities of neurons projecting from periaqueductal gray (PAG) to rostral ventromedial medulla (RVM) induced by a range of time courses of forced swim exposure. The moderate but not transitory or prolonged swim exposure induced a MOR-dependent analgesia, although all of these three stresses enhanced β-endorphin release. Selective deletion of MOR_GABA_ but not MOR_Glut_ clearly attenuated analgesia and blocked the enhancement of activities of PAG-RVM neurons induced by moderate swim exposure. Under transitory swim exposure, in contrast, selective deletion of MOR_Glut_ elicited an analgesia behavior via strengthening the activities of PAG-RVM neurons. These results indicate that MOR-dependent endogenous opioid signaling participates in nociceptive modulation in a wide range, not limited to moderate, of stress intensities. Endogenous activation of MOR_GABA_ exerts analgesia, whereas MOR_Glut_ produces antianalgesia. More importantly, with an increase of stress intensities, the efficiencies of MORs on nociception shifts from balance between MOR_Glut_ and MOR_GABA_ to biasing toward MOR_GABA_-mediated processes. Our results point to the cellular dynamic characteristics of MORs expressed in excitatory and inhibitory neurons in pain modulation under various stress intensities.

## Significance Statement

Mu-opioid receptors (MORs) are one of the major opioid receptors playing a critical role in stress-induced analgesia and are widely expressed in different types of neurons, but their potential roles expressed in glutamatergic (MOR_Glut_) and γ-aminobutyric acidergic (MOR_GABA_) neurons are poorly understood. This work clarifies the divergent roles of MOR_Glut_ and MOR_GABA_ in analgesia under various swim stress intensities. We demonstrate that MOR_GABA_ neurons are essential for stress-induced analgesia, whereas MOR_Glut_ neurons elicit an anti-analgesic-like response. The contributions of MOR_Glut_ and MOR_GABA_ neurons to analgesia depends on stress intensity, their opposite effects neutralizing each other under transitory stress and then biasing them toward MOR_GABA_ under moderate stress. This report appraises different roles for the MORs in these neuronal populations in modulating opioid-dependent stress-induced analgesia.

## Introduction

Stress-induced analgesia is an instinctive defensive reaction of mammals elicited by various acute stressful or negative stimuli, which can rapidly desensitize the pain response of the body and therefore dodge potential detriment in emergency circumstances like the fight-or-flight environment (Ferdousi and Finn, 2018). It is well demonstrated that stress-induced analgesia involves various neurotransmitters and neuropeptides, including opioids, cannabinoid, monoamine, GABA, and glutamate systems, leading to the activation of several intrinsic pain-suppressed mechanisms to produce analgesia. In particular, as critical pain regulators in CNS, endogenous opioid peptides (EOPs) energetically exert their antinociceptive effects by controlling the activation of the descending pain inhibitory pathway including the orbitofrontal cortex, the periaqueductal gray (PAG) of the midbrain, the rostral ventromedial medulla (RVM) of the brainstem, and the spinal cord dorsal horn in the processing of stress-induced analgesia (Gameiro et al., 2006; Butler and Finn, 2009).

Certain intensity of stress is required to induce opioid-dependent stress-induced analgesia. In general, moderate stress produces opioid analgesia and heavy stress produces nonopioid analgesia, whereas weak stress hardly produces any analgesic effect (Konecka et al., 1985; Miczek et al., 1985; Mogil et al., 1996). Several types of stressors in moderate intensity like restraint/immobilization, footshock or tailshock, forced swim, food and water deprivation, social isolation, and social conflict, that have been shown to trigger opioid-dependent stress-induced analgesia occur with naloxone (an opioid receptor antagonist) sensitivity (Konecka et al., 1985; Miczek et al., 1985; Menendez et al., 1993; Mogil et al., 1996; Larauche et al., 2012). Mu-opioid receptors (MORs), one of the four major opioid receptors encoded by *OPRM1* gene, play a critical role in opioid-dependent stress-induced analgesia (Fukunaga and Kishioka, 2000). Pharmacological and neurochemical studies have well demonstrated that either systemic or intracerebral and intrathecal administration of MORs blockage markedly reduced antinociception of rodents exposed in warm forced swim, intermittent footshock, and conditioned fear paradigms (Menendez et al., 1993; Mogil et al., 1996; Onodera et al., 2000; Wiedenmayer and Barr, 2000). Coincidentally, opioid-dependent stress-induced analgesia was significantly promoted by using selective MOR agonist systematically or intracerebrally (Calcagnetti et al., 1992; Vivian and Miczek, 1998). In addition, morphologic and biochemical studies have shown dense expressions of MORs in the core area of the descending pain inhibitory pathway (Stumm et al., 2004; Börner et al., 2007; Schnell and Wessendorf, 2008) and significant analgesic attenuation of opioid-dependent analgesia in MOR knock-out mice (Rubinstein et al., 1996; LaBuda et al., 2000; Contet et al., 2006), providing further proof in demonstrating the vital roles of MORs on opioid-dependent stress-induced analgesia at a molecular level.

The expression of MOR-dependent stress-induced analgesia is closely associated with the elevated β-endorphin (β-EP) levels. β-EP, one of EOPs that has the highest binding affinity for MORs and is synthesized immediately in the early period of stress (Konecka et al., 1985; Tsigos and Chrousos, 2002; Smith and Vale, 2006; Bodnar, 2013; Pilozzi et al., 2020). The significant rise of β-EP concentrations in blood plasma and several pain-related brain areas like hypothalamus, pituitary gland, and PAG have been clearly observed in various stress paradigms including food and water deprivation and social conflict (Konecka et al., 1985; Külling et al., 1988). Mice with low β-EP sensitivity exhibit a decrease in pain threshold compared with normal β-EP sensitivity when exposed to swim stress (Lim and Funder, 1983). In addition, MOR-dependent stress-induced analgesia also can be weakened by exogenous administration of β-EP neutralizing antibody (Pilozzi et al., 2020). The contribution of β-EP in MOR-dependent stress-induced analgesia was further confirmed by using gene knockout technology, while forced swim stress-induced MOR-dependent analgesia was significantly abolished in β-EP-deleted mice (Parikh et al., 2011). However, it is worth noting that, under certain stress intensities, the enhanced β-EP was not accompanied by the expression of stress-induced analgesia (Konecka et al., 1985), and the mechanisms underlining the mismatch between β-EP concentration and analgesia remain unclear.

In the CNS, MORs are predominately expressed in the axons, terminals, dendrites, and somata of GABAergic inhibitory interneurons spread over PAG and RVM. The activation of MORs is hypothesized to trigger the analgesic effects through suppression of inhibitory GABAergic inputs onto output neurons, which constitute the descending analgesic pathway, an indirect process of “GABA disinhibition” at the cellular level to activate the analgesic pathway (Drake and Milner, 2002; Lau and Vaughan, 2014). However, recent studies have also well clarified that in addition to GABAergic neurons, glutamatergic excitatory neurons also extensively expressed MORs throughout of the brain. Several pain-related areas, including prefrontal cortex, nucleus accumbens, thalamus, and PAG have considerable distribution of MORs in glutamatergic neurons (Schnell and Wessendorf, 2008; Zhang et al., 2020). This diversified distribution of MORs implies that the targets for β-EP to mediate MOR-dependent nociceptive modulation are not limited to GABAergic interneurons.

Given the diversified distribution of MORs throughout pain-related pathways, here we supposed that MORs expressed in subpopulations of cells within the pain modulatory circuit fundamentally mediate opioid-dependent stress-induced analgesia through distinct cellular bases when undergoing different intensities of stress stimuli. To test this hypothesis, we developed two lines of MOR-knock-out mice that lacked MORs specifically in GABAergic inhibitory mice (MOR_GABA_^−/−^) and glutamatergic mice (MOR_Glut_^−/−^), respectively (Fan et al., 2019; Shi et al., 2020). By using selectively MOR deletion mice, combined with the pharmacological blockage method and fiber photometry recording, we exhibited a MOR-dependent stress-induced analgesia induced by different intensities of swim stress MOR_GABA_ and MOR_Glut_ deletion mice. Furthermore, the contributions of MOR_GABA_ and MOR_Glut_ on calcium activities of neurons projecting from PAG to RVM in stress-induced analgesia were further investigated.

## Materials and Methods

### Animals

All experiments with animals were performed in accordance with the Chinese Council on Animal Care and were approved by the Animal Care Committee of Shaanxi Normal University. One hundred and eight adult male C57BL/6J mice (from Model Animal Research Center, Nanjing University, Nanjing, People’s Republic of China), 72 MOR mutant mice, and 48 littermate controls (Beijing Biocytogen) that were 10–12 weeks of age were used in this study. Seventy-two C57BL/6J mice were used for behavioral pharmacology, 24 were prepared for β-EP ELISA test, and others were administered a GCaMP adeno-associated virus (AAV) to serve as controls on fiber photometry recording. For MOR mutant mice, 24 were devoted to fiber photometry recording, others and 48 of their littermate controls were used for behavioral tests. Animal grouping in each part of the experiment was stated individually in the Results section. All animals were housed in groups of four in individually ventilated cages and maintained at 22 ± 2°C and 55 ± 5% relative humidity under a normal 12 h light/dark cycle. Behavior experiments were conducted between 9:00 A.M. and 11:00 A.M. The animals were habituated for 5 d to acclimate to the environment and apparatus before behavioral experiments.

Mutant mouse lines were produced as below. Mice specifically lacking MORs in MOR_GABA_^−/−^ neurons or MOR_Glut_^−/−^ neurons were generated by crossing *Oprm1* floxed mice to *Gad2iCreERT2* mice or *vGlut1iCreERT2* mice, respectively. The adult *Oprm1^loxP/loxP^:Gad2iCreERT2* or *Oprm1^loxP/loxP^:vGlut1iCreERT2* mice were treated with tamoxifen (2 mg/d, i.p.; Sigma-Aldrich) for 7 consecutive days to induce the deletion of MORs and were used for the experiments 2 weeks after the last injection. The littermates of *Oprm1flox/flox:CreERT2*^−/−^ mice receiving similar tamoxifen treatment were taken as their control (MOR_GABA_^+/+^, MOR_Glut_^+/+^). To verify the deletion of MORs in the mutant mice, fluorescence *in situ* hybridization was used. The MOR_GABA_ deletions were verified in PAG (see Fig. 2*A*,*B*) and hippocampus (Shi et al., 2020). While PAG is lacking *vGlut1*-positive neurons (Soiza-Reilly and Commons, 2011), MOR_Glut_ deletions were verified in hippocampus (Shi et al., 2020) and anterior cingulate cortex (ACC), a major brain area sending *vGlut1*-positive projections to PAG (see Fig. 2*C*,*D*). The efficiency of the cell-type deletion of MORs was calculated (Fig. 2*E*,*F*).

### Forced swim stress

Animals were placed individually in a cylindrical plastic container 30 cm in diameter and 45 cm in height. The level of the water ranged from 30 cm above the bottom of cylindrical plastic so as to avoid the escape of mice. The water temperature was maintained approximately 32 ± 1°C in all experiments. The forced swim stress was induced by placing animals on the surface of the water for 1.5, 3, or 6 min. After stress, animals were carefully taken out from the plastic container and the surface moisture of skin was erased within 60 s to ensure that the skin condition was in accord with that of the prestress state.

### Analgesia test

To examine the stress-induced analgesia, the tail-flick test was conducted before and immediately after swim exposure. Briefly, the animals were mildly restricted by a restrainer with their tail positioned in an apparatus (type YLS-12A, Shanghai Bio-will Co, Ltd.) for radiant heat stimulation on the surface of the tail. The tail-flick latency was defined as the time interval between the application of the radiant heat stimulation onto the tail and the abrupt removal of the tail from the nociceptive stimulus. The infrared wavelength of the radiant heat source was set at 805 nm, and the output power was set at 28 W, so that the baseline tail-flick latencies of the mice were controlled between 2 and 4 s. The cutoff time was set at 10 s to avoid tissue damage. For each animal, two sessions of tail-flick test, containing tail-flick latency before and after stress latency were conducted. Latency before stress was detected 120 min before swim stress, and latency after stress was tested immediately when the surface moisture of animal skin was dried after swim stress. Three measurements (with ∼60 s intervals) were taken from each session then averaged to determine tail-flick latency. Results were expressed as the percentage maximum possible effect (MPE), which was calculated as follows: MPE % = (poststress latency – prestress latency)/(cutoff latency – prestress latency) × 100%.

### Drugs

Saline (0.9%) and β-funaltrexamine [β-FNA (a MOR-specific antagonist); Sigma-Aldrich] were used to identify the influence of forced swim intensities on MOR-dependent forced swim stress-induced analgesia. Mice were treated with physiological saline or β-FNA dissolved in saline through subcutaneous injection with a dose of 40 mg/kg body weight 24 h before the stress and analgesia test. The volume of injection was set at 10 ml/kg.

### Determination of serumal β-EP levels by ELISA

To detect the connection between swim stress intensities and β-EP levels, whole-blood samples were collected and let it stand for ∼20 h at 4°C, then the upper serum of blood was carefully sampled and stored in aliquot at −20°C before ELISA testing. At the time of detection, β-EP levels of serum were detected by using mouse β-EP ELISA Kit (lot JL10523-48T, J&L Biological).

### AAV injection surgery

Animals were anesthetized with 4% isoflurane by immobilizing a chamber for small animals and then fixed with brain solid positioner with nonpuncturing ear bars. The 1% isoflurane was continuously delivered to nasopharynx of animals during surgery to maintain the anesthetic effect. Both eyes were smeared with eye ointment to avoid strong light stimulation and eye drying. After head hair was shaved and cranial dura mater was cut, retrograde transport GCaMP6f virus rAAV2/R-hsyn-GCaMP6f-WPRE-hGH-pA (300 nl/injection, 2.0 × 10^12^ copies/ml; BrainVTA) was injected into RVM of C57BL/6J, MOR_GABA_^−/−^, or MOR_Glut_^−/−^ mice by using a Hamilton microsyringe. To ensure the authenticity of GCaMP recording, rAAV2/R-hsyn-EYFP-WPRE-pA (300 nl/injection, 2.0 × 10^12^ copies/ml; BrainVTA) was also injected into C57BL/6J mice as a control. The injection site was used according to the mouse brain map: anteroposterior (AP), −5.80 mm; mediolateral (ML), 0 mm; dorsoventral (DV), −5.80 mm. After virus injection, the microsyringe was reserved *in situ* for 10 min, then the optical fiber ferrule was unilaterally inserted into PAG, according to the sites (AP, −4.80 mm; ML, 0.35 mm; DV, −2.80 mm) so that the calcium activities of neurons projecting from PAG to RVM could be monitored. Glass sonomer cement was finally smeared within the junction between the skull of animals and the optical fiber ferrule so as to strengthen the stability of the optical fiber ferrule. After surgery, all animals were housed at least 3 weeks before subsequent experiments.

### Fiber photometry recording

To account for the calcium activities of neurons projecting from PAG to RVM, tail immersion of hot water was used as previously described (Moriya et al., 2020; Zhang et al., 2020). Animals were immobilized in the same restricted apparatus used for the tail-flick test with their tail exposed to receive the tail stimulation; and recording sessions of animals were also in sync with the tail-flick test, with basic calcium activities detected 120 min before stress, and poststress calcium activities recorded immediately when the surface moisture of animal skin was dried after swim stress. In the processing of fiber photometry recording, the root of the animal tail was immersed in 55°C hot water for 2 s to elicit calcium activities of projecting neurons, and 25°C water was set as its control. Each animal treated tail immersion three times with an interval of >60 s. In each stimulation, the “baseline windows” were defined as 5 s before each tail immersion, and the averaged fluorescence signal intensities within this time were denoted as *F*_0_. The “event windows” were defined as 5 s after stimulus, and the averaged fluorescence signal intensities within this time were denoted as *F*_s_. The environmental and systematic noises were recorded after disconnecting the tip of the fiber from an implanted ceramic ferrule and blocking any optic input; these parts of averaged fluorescence signal intensities were denoted as *F*_n_. The dynamics of the fluorescence signal intensities in stimuli were denoted as △*F*/*F* and calculated by computational formula: △*F*/*F* = *F*_s_ – *F*_0_/*F*_0_ – *F*_n_. The measuring result of each mice was the average of three times the calculated △*F*/*F*. The sampling frequency was set at 50 Hz. Data were transferred and presented in MATLAB 2014.

### Tissue preparation and fluorescence imaging

After recording, fluorescence imaging was used to detect the infection efficiency of AAV. Mice were deeply anesthetized with 25% urethane and transcardially perfused with normal saline and 4% dissolved in paraformaldehyde (PFA) and PBS successively. The brains were then taken out and postfixed in the same PFA PBS solution. Next, the brains were perfused with running water for at least 4 h and were soaked in a 30% sucrose PBS solution for 48 h at 4°C. Sections including PAG were finally sliced by using a cryostat (model CM 1950, Leica Biosystems) and imaged by fluorescence microscope (Zeiss USA).

### Fluorescence *in situ* hybridization with RNAscope

The deletion of MORs in mutant mice was verified by fluorescence *in situ* hybridization assay (Fan et al., 2019; Shi et al., 2020). Briefly, mice were deeply anesthetized with isoflurane and killed by perfusion with 4°C saline (0.9%) within 5 min. The brains were quickly removed from the skulls and frozen on dry ice, and then embedded in OCT (catalog Tissue-Tek 4583, Sakura Finetek USA). Fresh frozen sections (16 μm) were made coronally with a freezing microtome (model CM1950, Leica Biosystems) and thaw mounted onto Superfrost Plus Microscope Slides (Thermo Fisher Scientific). The sections were fixed in 4% PFA for 60 min at 4°C before being dehydrated using graded ethanol (50%, 70%, and 100%) at room temperature for 5 min each and finally air dried. The sections were incubated with hydrogen dioxide for 10 min and subsequently pretreated with protease IV for 15 min. The probes for Oprm1 (16 synthetic oligonucleotides complementary to the nucleotide sequence 590–1458 of Oprm1), Gad2 (catalog #39371) and vglut1 (catalog #416631) were provided by Advanced Cell Diagnostics (ACD) and conjugated to Atto 594 and Atto 488, respectively. The procedure for *in situ* detection was performed using RNAscope Multiplex Fluorescent Reagent Kit version 2 (catalog #323100, ACD) according to the manufacturer instructions for fresh frozen tissue. After being heated with a HybEZTM oven (ACD) for 2 h, slides were mounted with the ProLong Gold Antifade Mountant (catalog #P10144, Thermo Fisher Scientific). Confocal images were captured with a fluorescence microscope (Zeiss), and cells with positive labeling were counted.

### Statistical analysis

Data were presented as the mean ± SEM and were analyzed by using SPSS 22.0 software. Paired Student’s *t* test, unpaired Student’s *t* test, one-way ANOVA, and two-way ANOVA were used, as stated individually in the Results section. The one-way ANOVAs were followed by a Dunnett’s multiple-comparisons test. The two-way ANOVAs were followed by Sidak’s multiple-comparisons test. The level of significance was set at *p *<* *0.05 in all experiments.

## Results

### The involvement of β-EP and MORs in opioid-dependent stress-induced analgesia

**Figure 1.**
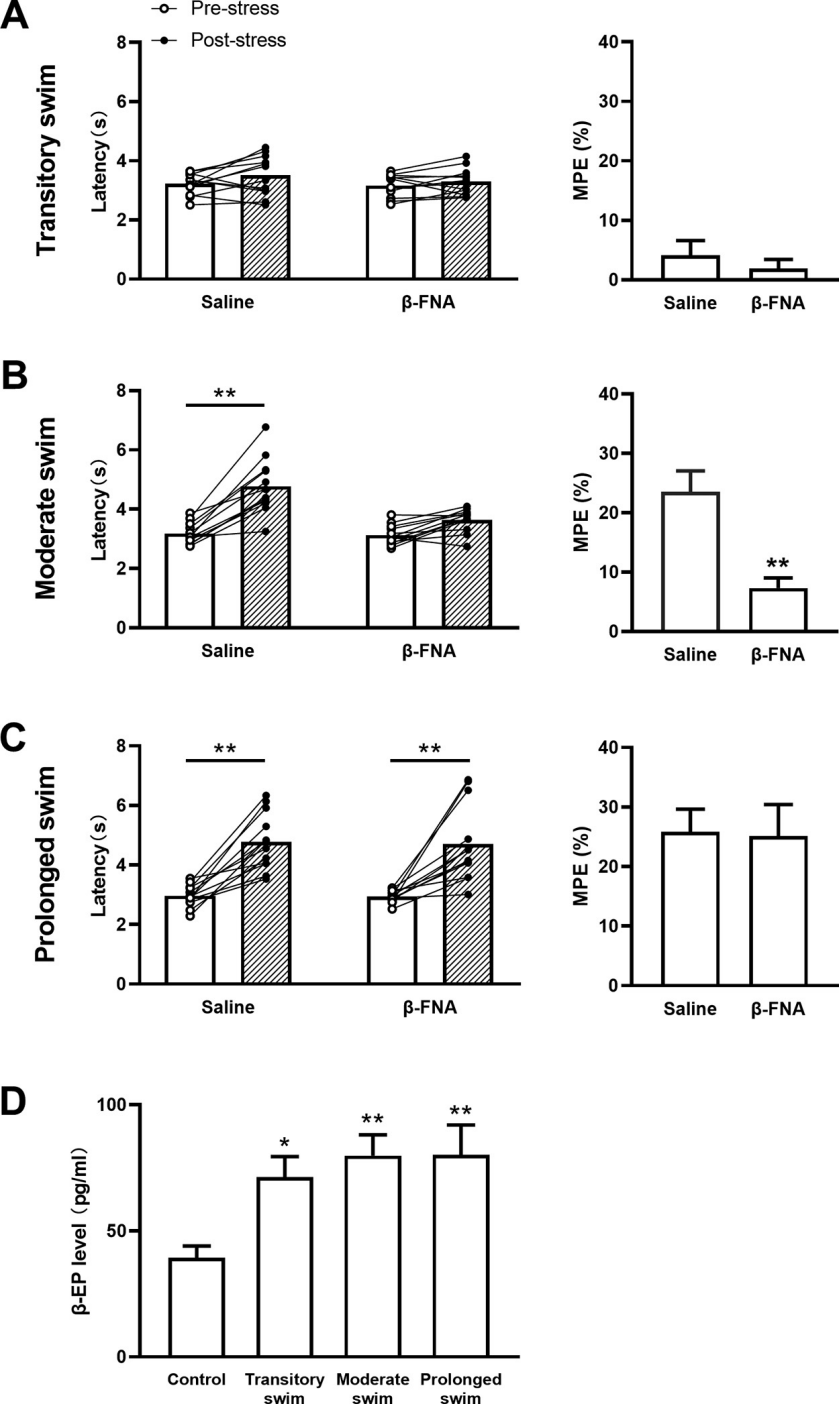
The involvement of β-EP and MORs on opioid-dependent stress-induced analgesia. ***A–C***, Left, Tail-flick latency under transitory swim (***A***), moderate swim (***B***), or prolonged swim (***C***) exposure in mice pretreated with saline or β-FNA; *n* = 12 for each group, **p <* *0.01. Right, Equated MPE percentage from left groups, with data shown as the mean ± SEM. **p <* *0.01 versus saline. ***D***, β-EP levels in serum analysis using ELISA. *n* = 6 for each group, data are shown as mean ± SEM. *p <* *0.05; **p <* *0.01 versus control.

**Figure 2.**
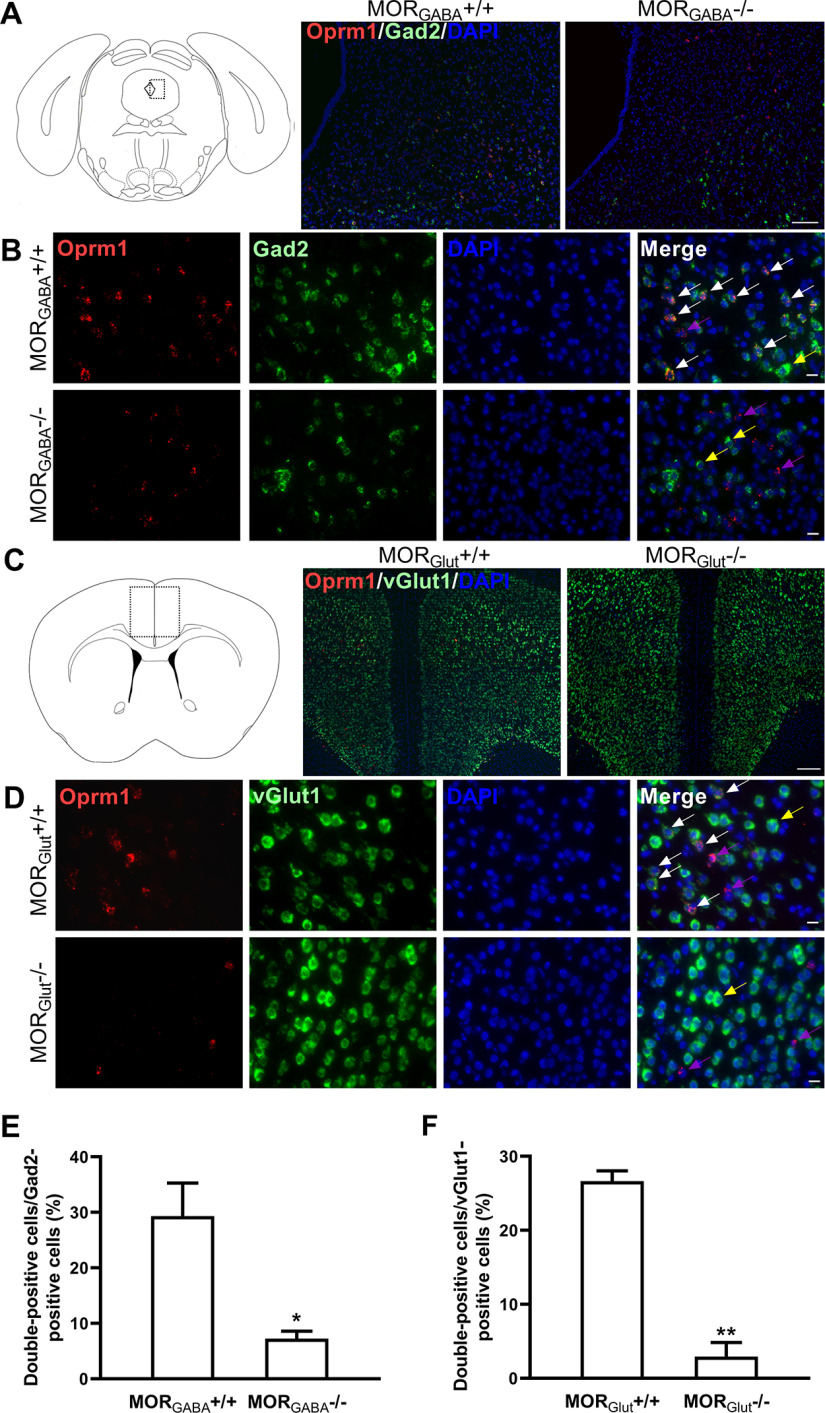
Generation of conditional knock-out mice specifically lacking MORs on glutamatergic and GABAergic neurons. ***A***, Schematic of *in situ* hybridization for *Oprm1* mRNA and *GAD2* mRNA in the PAG areas in MOR_GABA_^+/+^ and MOR_GABA_^−/−^ mice. The nucleus is stained in blue (DAPI), *GAD2* mRNA is stained in green, and *Oprm1* mRNA is stained in red. Scale bar, 100 μm. ***B***, Higher-magnification images of the fields in the PAG areas in MOR_GABA_^+/+^ and MOR_GABA_^−/−^ mice. The white arrowhead indicates a double-labeled cell with *Oprm1* mRNA and *GAD2* mRNA; the purple arrowheads represent *Oprm1* mRNA localization in *GAD2*-negative cells; and the yellow arrowheads represent *GAD2*-positive cells without *Oprm1* mRNA. Scale bar, 20 μm. ***C***, Schematic of *in situ* hybridization for *Oprm1* mRNA and *vGlut1* mRNA in the ACC areas in MOR_Glut_^+/+^ and MOR_Glut_^−/−^ mice. The nucleus is stained in blue (DAPI), *vGlut1* mRNA is stained in green, and *Oprm1* mRNA is stained in red. Scale bar, 200 μm. ***D***, Higher-magnification images of the fields in the ACC areas in MOR_Glut_^+/+^ and MOR_Glut_^−/−^ mice. The white arrowhead indicates a double-labeled cell with *Oprm1* mRNA and *vGlut1* mRNA; the purple arrowheads represent *Oprm1* mRNA localization in *vGlut1*-negative cells; and the yellow arrowheads represent *vGlut1*-positive cells without *Oprm1* mRNA. Scale bar, 20 μm. ***E***, Quantitative analysis of the percentage of double-positive neurons (*Oprm1* and *GAD2*) against the *GAD2*-positive neurons. *p <* *0.05 versus MOR_GABA_^+/+^, unpaired *t* test (*n* = 3 in each group). ***F***, Quantitative analysis of the percentage of double-positive neurons (*Oprm1* and *vGlut1*) against the *vGlut1*-positive neurons. **p <* *0.01 versus MOR_Glut_^+/+^, unpaired *t* test (*n* = 3 in each group).

Forced swim stress can steadily induce analgesic effects. Previous studies have shown that a 3 min swim exposure pattern in 32°C water causes opioid-dependent antinociception (Mogil et al., 1996), but the roles of MORs in this procedure are still unknown. To explore the contributions of MORs on forced swim stress-induced analgesia, β-FNA, a highly selective and irreversible MOR antagonist, was applied 24 h before the stress (Hayes et al., 1985; Banks, 2015). We examined the effect of transitory (1.5 min), moderate (3 min), and prolonged (6 min) forced swim stress on pain behaviors. Consistent with a previous study (Mogil et al., 1996), no analgesic effect was found in both saline-pretreated mice and β-FNA-pretreated mice after transitory swim exposure (drug treatment: *F*_(1,11)_ = 0.7524, *p *=* *0.4042; stress: *F*_(1,11)_ = 3.668, *p *=* *0.0818; interaction: *F*_(1,11)_ = 0.7527, *p *=* *0.4029; two-way ANOVA; Fig. 1*A*); MPE percentage further revealed that β-FNA made little impact on pain behavior (*p *=* *0.6316, unpaired Student’s *t* test; Fig. 1*A*, right). However, saline-pretreated mice showed an obvious analgesia under moderate swim exposure, which could be dramatically attenuated by β-FNA pretreatment shown in both tail-flick latencies (drug treatment: *F*_(1,11)_ = 8.396, *p *=* *0.0145; stress: *F*_(1,11)_ = 95.08, *p *=* *0.0000; interaction: *F*_(1,11)_ = 13.82, *p *=* *0.0034; two-way ANOVA; Fig. 2*B*) and MPE percentage (*p *=* *0.0004, unpaired Student’s *t* test; Fig. 1*B*, right). In addition, a significant analgesic effect was shown in both saline-pretreated mice under prolonged swim exposure (drug treatment: *F*_(1,11)_ = 0.04057, *p *=* *0.8440; stress: *F*_(1,11)_ = 55.39, *p *=* *0.0000; interaction: *F*_(1,11)_ = 0.0084, *p *=* *0.9204; two-way ANOVA; Fig. 1*C*), and MPE percentage showed insignificance between two groups (*p *=* *0.8922, unpaired Student’s *t* test; Fig. 1*C*, right). These results indicate a secondary analgesic role of MORs in prolonged swim stress.

To test whether MORs are endogenously activated by EOPs after stress, the levels of β-EP, an EOP with high binding affinity for MORs in forced swim stress (Parikh et al., 2011), was tested by ELISA. Surprisingly, remarkable differences of serum β-EP levels were exhibited among distinct stress intensities (*F*_(3,20)_ = 4.982, *p *=* *0.0096, one-way ANOVA; Fig. 1*D*), and significant increases were found in all of the forced swim exposures compared with control mice (*p *=* *0.0429 for transitory swim; *p *=* *0.0094 for moderate swim; *p *=* *0.0090 for prolonged swim), though there was no analgesic effect following transitory swim exposure (Fig. 1*A*). Therefore, our results indicate that all transitory, moderate, and prolonged swim exposures can activate the endogenous opioid system, but only moderate swim exposure induces the expression of MOR-dependent analgesic behavior.

### The contributions of MORs expressed in glutamatergic and GABAergic neurons to stress-induced analgesia

The MORs are expressed not only in inhibitory neurons but also excitatory neurons, and both of them are involved in pain modulation. To address the distinct effects of MORs in glutamatergic (MOR_Glut_) and GABAergic (MOR_GABA_) neurons on analgesia under transitory or moderate swim exposure, two mice lines specifically lacking, respectively, MOR_GABA_ (MOR_GABA_^−/−^) and MOR_Glut_ (MOR_Glut_^−/−^) were used. The deletion of MORs in the mutant mice was verified by using RNAscope (Fig. 2). No significant difference in the basal tail-flick latency was detected between mice with MOR deletions and their wild-type littermates, respectively (*p *>* *0.05, unpaired Student’s *t* tests). However, a diverse modulation of MORs on the analgesic effect related to stress intensities were found in those mouse lines.

**Figure 3.**
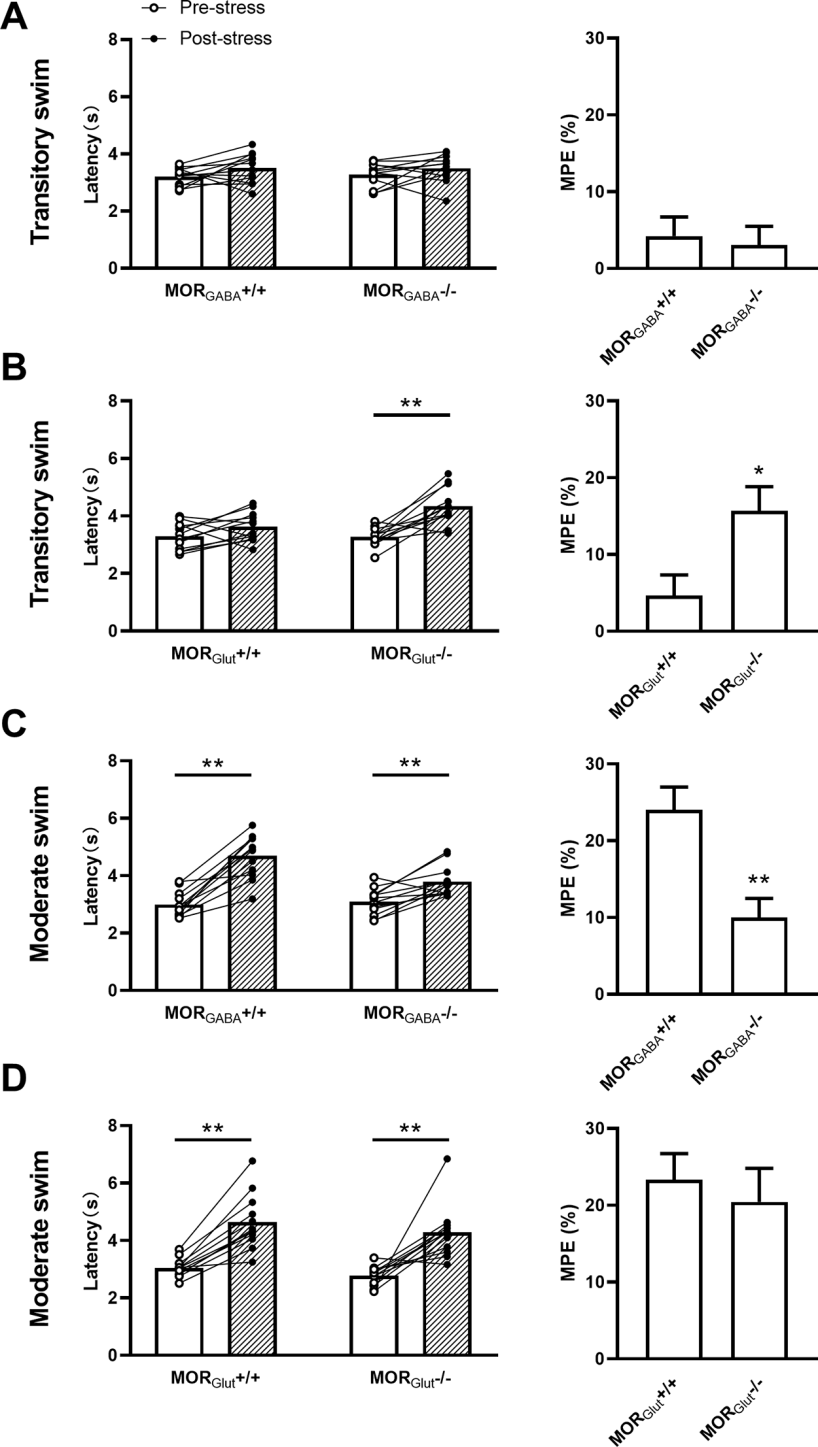
***A–D***, The contributions of MORs expressed in glutamatergic (***B***, ***D***) and GABAergic (***A***, ***C***) neurons to stress-induced analgesia under transitory (***A***, ***B***) or moderate (***C***, ***D***) swim exposure. Left, Tail-flick latency. Right, Equated MPE percentage; *n* = 12 for each group; data are shown as the mean ± SEM. **p <* *0.01.

After transitory swim exposure, both MOR_GABA_^−/−^ mice and their littermates still exhibited nondetectable change of the tail-flick latencies (genotype: *F*_(1,11)_ = 0.0705, *p *=* *0.7955; stress: *F*_(1,11)_ = 2.959, *p *=* *0.1134; interaction: *F*_(1,11)_ = 0.2043, *p *=* *0.6601; two-way ANOVA; Fig. 3*A*, left) and MPE percentage (*p *=* *0.7433, unpaired Student’s *t* test; Fig. 3*A*, right). However, the tail-flick latencies of MOR_Glut_^−/−^ mice were significantly increased after stress, whereas no significant changes were seen in their wild-type littermates (genotype: *F*_(1,11)_ = 4.874, *p *=* *0.0494; stress: *F*_(1,11)_ = 20.61, *p *=* *0.0008; interaction: *F*_(1,11)_ = 9.890, *p *=* *0.0093; two-way ANOVA; Fig. 3*B*, left). Meanwhile, the MPE percentage of these two groups displayed a significant difference (*p *=* *0.0059, unpaired Student’s *t* test; Fig. 3*B*, right), implying the involvement of MOR_Glut_ on pain mediation under transitory swim exposure.

Under moderate swim exposure, although the tail-flick latencies of both MOR_GABA_^−/−^ mice and their littermates showed significant increase after stress, the increased magnitude of latency from MOR_GABA_^−/−^ mice was much inhibited, compared with their littermates (genotype: *F*_(1,11)_ = 4.118, *p *=* *0.0673; stress: *F*_(1,11)_ = 59.37, *p *=* *0.0000; interaction: *F*_(1,11)_ = 20.76, *p *=* *0.0008; two-way ANOVA; Fig. 3*C*, left). The MPE percentage also exhibited a significant difference between groups (*p *=* *0.0023, unpaired Student’s *t* test; Fig. 3*C*, right), although the tail-flick latencies of both genotype mice were significantly increased. In contrast, the tail-flick latencies of both MOR_Glut_^−/−^ mice and their littermates showed almost the same degree of increase after stress (genotype: *F*_(1,11)_ = 3.005, *p *=* *0.1109; stress: *F*_(1,11)_ = 51.37, *p *=* *0.0000; interaction: *F*_(1,11)_ = 0.0578, *p *=* *0.8144; two-way ANOVA; Fig. 3*D*, left), and the MPE percentage also exhibited insignificant differences between the groups (*p *=* *0.6357, unpaired Student’s *t* test; Fig. 3*D*, right). These results indicate a predominant contribution of MOR_GABA_ to analgesia under moderate swim exposure, though their additional analgesic components involved in this stress-induced analgesia paradigm could not be excluded.

Thus, our results reveal an opposite modulation of the MOR_Glut_ and MOR_GABA_ on stress-induced analgesia. The activation of MOR_GABA_ produces analgesia, whereas MOR_Glut_ elicits anti-analgesic-like effects. Furthermore, the functional balance between MOR_Glut_ and MOR_GABA_ on pain modulation could be broken depending on stress intensity.

### Activation of PAG–RVM-projecting neurons responding to thermal noxious stimulus

**Figure 4.**
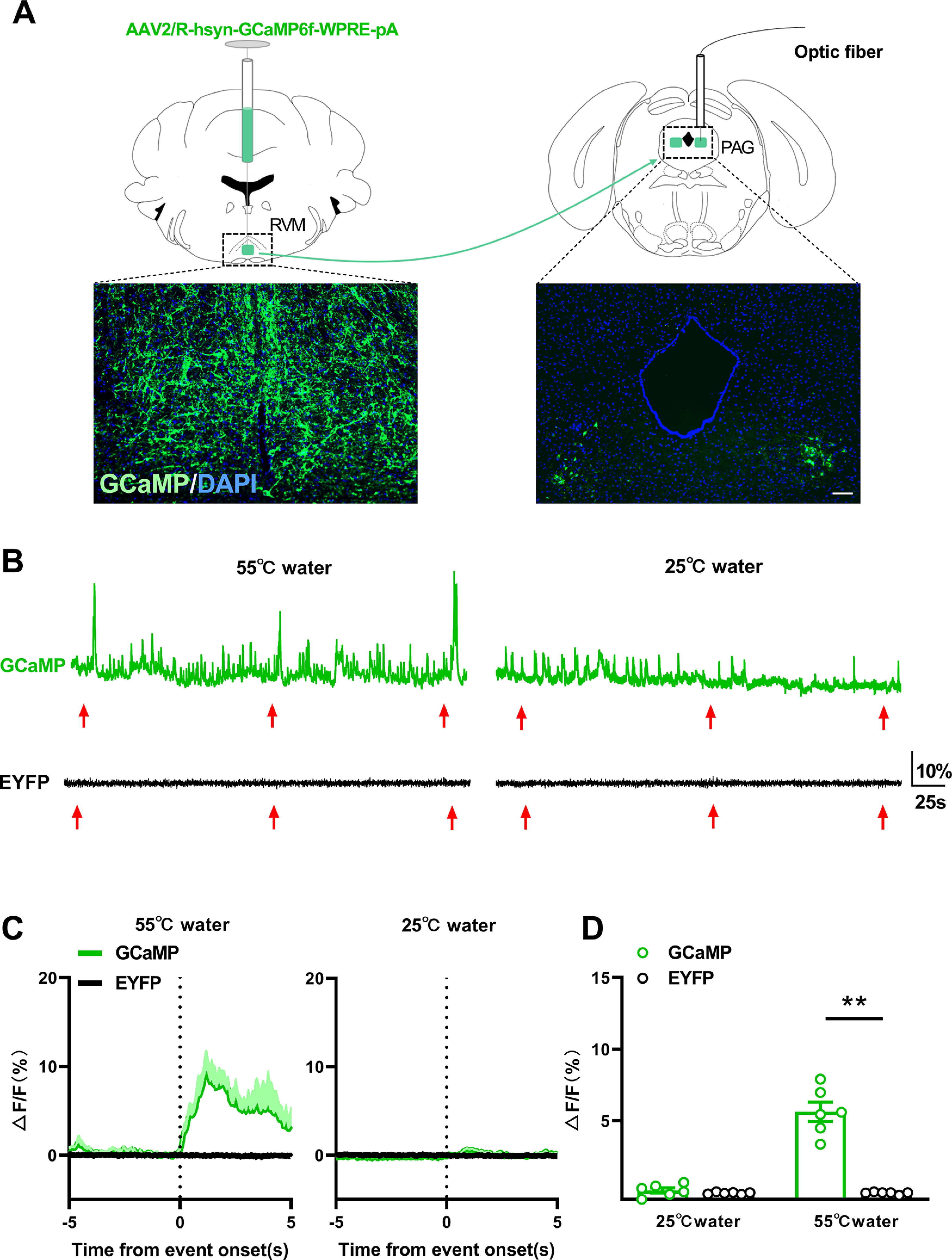
The responses of PAG-RVM neurons to thermal stimulus. ***A***, Schematic representation of the virus injection sites and optic fiber insertion sites, and corresponding schematic of GCaMP expression on RVM and PAG. Scale bar, 100 μm. ***B***, Typically representative photometry traces from GCaMP (top) or EYFP (bottom) mouse relative to the onset in response to 55°C (left) and 25°C (right) water tail immersion. Each red arrow represents an event (tail immersion stimulus). ***C***, Time course of the averaged fluorescence signal change in response to tail immersion. Light-colored shadow indicates the SEM; *n* = 6 for each group. ***D***, Comparison of the averaged fluorescence signal change between EYFP and GCaMP groups during the onset period (0–5 s) for each stimulation. **p<0.01.

In the descending pain inhibitory pathway, the activation of neurons projecting from PAG to RVM play a crucial role for stress-induced analgesia. To investigate the impacts of swim exposure on the activities of PAG–RVM-projecting neurons, *in vivo* calcium signals of those cells were tested by injecting the retrograde transport GCaMP6f virus or virus enhanced yellow fluorescent protein (EYFP; as control) into RVM, which axoplasmic transported from axons in RVM to somata in PAG (Fig. 4*A*). The availability of GCaMP6f expressions in RVM and PAG were confirmed by histologic method (Fig. 4*A*).

Previous studies have well demonstrated that the pain modulatory projections have the ability to respond to and tackle nociceptive information (Samineni et al., 2019; Huang et al., 2020; Moriya et al., 2020; Zhang et al., 2020). To verify that the nociceptive information has the ability to activate PAG–RVM-projecting neurons, their responses to thermal noxious stimulus (55°C water) or thermal innocuous stimulus (25°C water) were monitored by recording the fluorescence intensities.

On the background of spontaneous fluorescence signals, which indicated that there are tonic activities of PAG–RVM neurons under the basal condition, mice treated with GCaMP virus exhibited a large magnitude of fluorescence signals in response to thermal noxious stimulus but not to thermal innocuous stimulus (Fig. 4*B*,*C*). No fluorescence responses to either of the two stimuli were observed in EYFP mice, indicating that these detectable fluorescence signals during thermal noxious stimulation are not because of movement artifact (Fig. 4*B*,*C*). The analysis (two-way ANOVA; Fig. 4*D*) of averaged fluorescence responses during event windows (0–5 s after thermal stimulus) in each session revealed a significant difference of virus (*F*_(1,5)_ = 93.63, *p *=* *0.0002) and stimulus (*F*_(1,5)_ = 115.5, *p *=* *0.0001), and a significant interaction between virus and stimulus (*F*_(1,5)_ = 70.68, *p *=* *0.0004). Subsequent Sidak’s multiple-comparisons test revealed that there was a significant difference of fluorescence intensities between thermal noxious and innocuous stimuli in GCaMP mice (*p *=* *0.0004), and a significant difference between GCaMP and EYFP mice under 55°C water stimulus (*p *=* *0.0003), indicating that PAG-RVM neurons are specifically involved in the handling of thermal noxious information.

### The effects of MOR_Glut_ and MOR_GABA_ on activities of PAG–RVM-projecting neurons under different stress intensities

**Figure 5.**
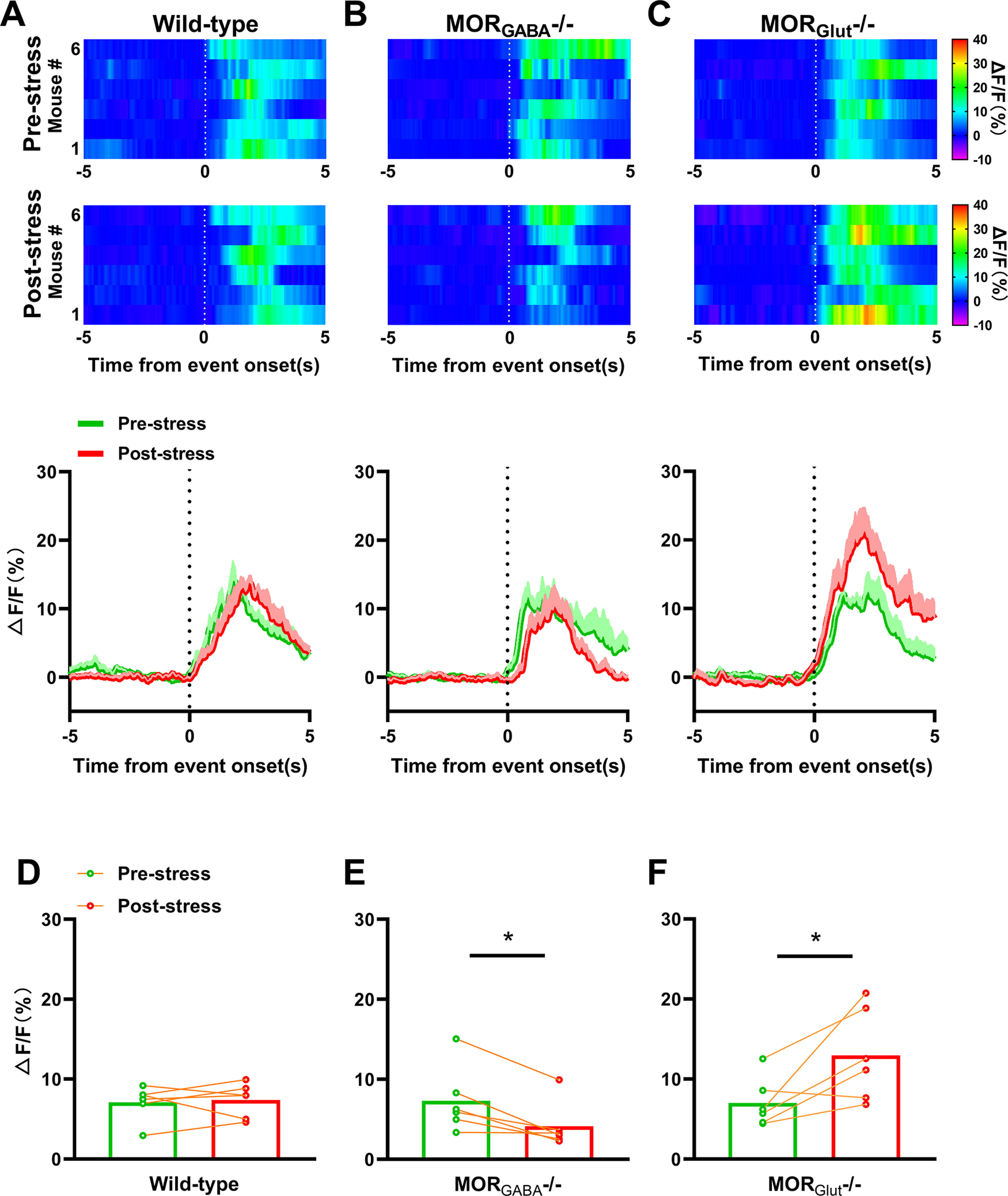
Changes in fluorescence intensities of PAG-RVM neurons induced by thermal stimulus under transitory swim exposure. ***A–C***, Top, Heat map of averaged fluorescence dynamics of GCaMP relative to the onset prestress and poststress in response to 55°C water tail immersion on wild-type, MOR_GABA_^−/−^, or MOR_Glut_^−/−^ mice; *n* = 6 for each group. Bottom, Time course of the averaged fluorescence intensities prestress and poststress on wild-type, MOR_GABA_^−/−^, or MOR_Glut_^−/−^ groups. ***D–F***, Comparison of the averaged fluorescence intensities between prestress and poststress during the onset period (0–5 s) for each stimulation on wild-type, MOR_GABA_^−/−^, and MOR_Glut_^−/−^ mice. *p < 0.05.

Next, we investigated the influence of stress on activities of PAG-RVM neurons responding to thermal stimulus. Consistent with behavioral results, after transitory swim exposure, fluorescence intensities of PAG–RVM-projecting neurons responding to thermal noxious stimulus in wild-type mice were almost unaltered, compared with that of prestress (Fig. 5*A*). In contrast, MOR_Glut_^−/−^ mice exhibited an obvious enhancement of fluorescence intensities after stress (Fig. 5*C*), and, surprisingly, fluorescence intensities of MOR_GABA_^−/−^ mice were decreased in poststress (Fig. 5*B*) without change of behavioral performance (Fig. 3*A*). The further analysis of averaged fluorescence intensities during event windows revealed an insignificant difference between prestress and poststress in wild-type mice (*p *=* *0.7404, paired Student’s *t* test; Fig. 5*D*), and a significant decrease in MOR_GABA_^−/−^ mice (*p *=* *0.0112, paired Student’s *t* test; Fig. 5*E*), but a significant increase in MOR_Glut_^−/−^ mice (*p *=* *0.0379, paired Student’s *t* test; Fig. 5*F*), respectively. These results suggest that MORs expressed in both GABAergic neurons and glutamatergic neurons are indeed involved in pain modulation under transitory swim exposure, and exhibit opposite effects on activities of PAG-RVM neurons in the descending pain inhibitory pathway.

**Figure 6.**
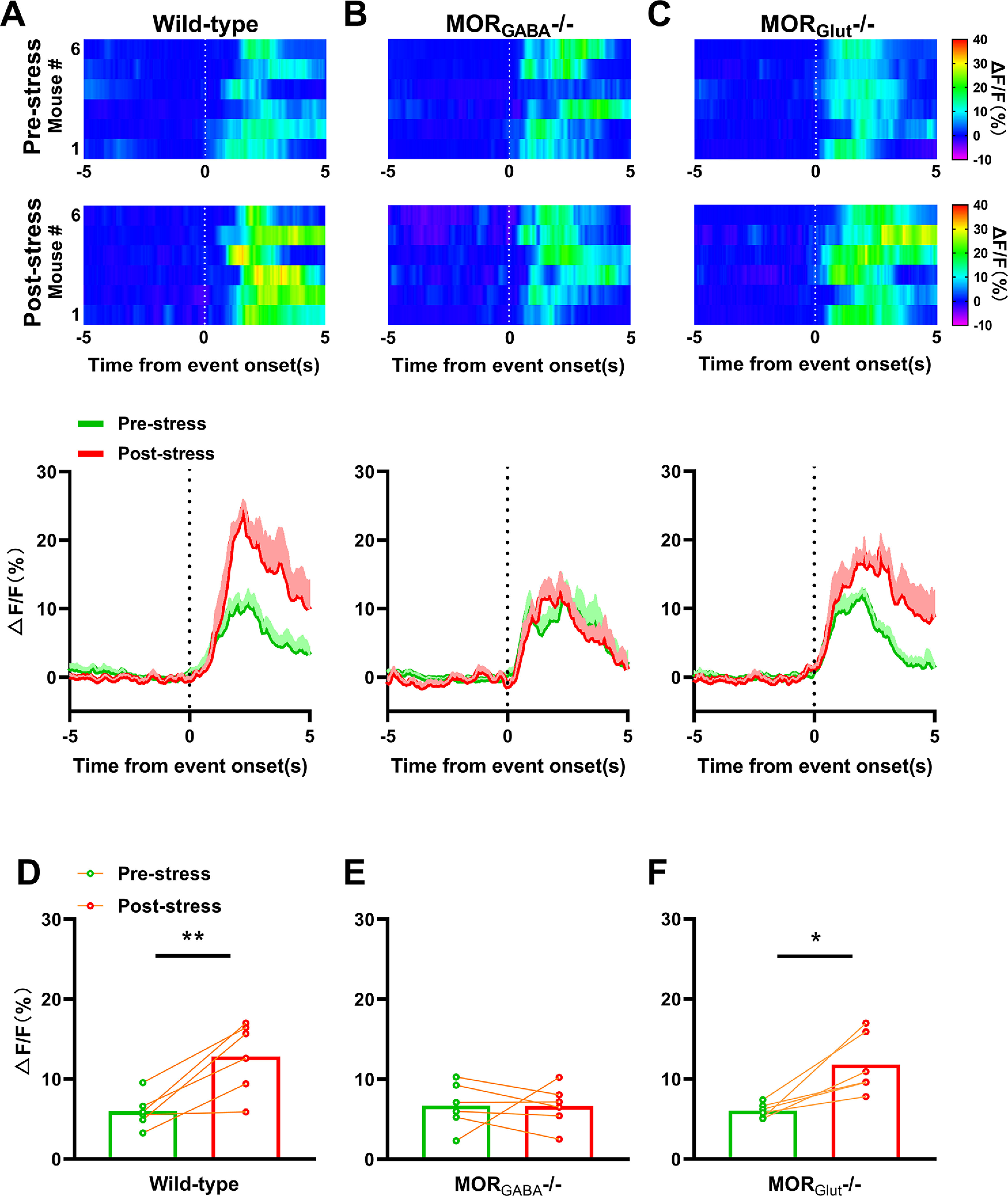
Changes in the fluorescence intensities of PAG-RVM neurons induced by thermal stimulus under moderate swim exposure. ***A–C***, Top, Heat map of averaged fluorescence dynamics of GCaMP relative to the onset prestress and poststress in response to 55°C water tail immersion on wild-type, MOR_GABA_^−/−^, or MOR_Glut_^−/−^ mice; *n* = 6 for each group. Bottom, Time course of the averaged fluorescence intensities prestress and poststress on wild-type, MOR_GABA_^−/−^, or MOR_Glut_^−/−^ groups. ***D–F***, Comparison of the averaged fluorescence intensities between prestress and poststress during the onset period (0–5 s) for each stimulation on wild-type, MOR_GABA_^−/−^, and MOR_Glut_^−/−^ mice. *p < 0.05, **p < 0.01.

On the other hand, after moderate swim exposure, the response of PAG-RVM neurons to thermal stimulus from both wild-type and MOR_Glut_^−/−^ mice increased remarkably (Fig. 6*A*,*C*). However, this increased response was lacking in MOR_GABA_^−/−^ mice (Fig. 6*B*). The analysis of averaged fluorescence intensities during event windows revealed that there were significant increases in fluorescence signal in both wild-type mice (*p *=* *0.0079, paired Student’s *t* test; Fig. 6*D*) and MOR_Glut_^−/−^ mice (*p *=* *0.0138, paired Student’s *t* tests; Fig. 6*F*), but not in MOR_GABA_^−/−^ mice (*p *=* *0.9799, paired Student’s *t* test; Fig. 6*E*) after stress. These results imply that, under moderate swim exposure, the enhanced activities of PAG-RVM neurons from wild-type mice can mainly be attributed to the activation of MOR_GABA_, which covers the opposite effects of MOR_Glut_ to inhibit activities of PAG-RVM neurons.

## Discussion

Here, we illuminated the opposite contributions of MOR_Glut_ and MOR_GABA_ on analgesia induced by various intensities of forced swim stress. The activation of MOR_GABA_ produces analgesia, whereas MOR_Glut_ tends to elicit anti-analgesic-like effects. Furthermore, the effects of these two MORs in distinct subpopulations of neurons on pain modulation showed a dynamic character in responding to stress intensity: with progressive enhancement of stress intensity, the efficiencies of MORs on nociception shifts from balance between MOR_Glut_ and MOR_GABA_ to biasing toward MOR_GABA_-mediated disinhibition, and finally to loss of efficacy.

**Figure 7.**
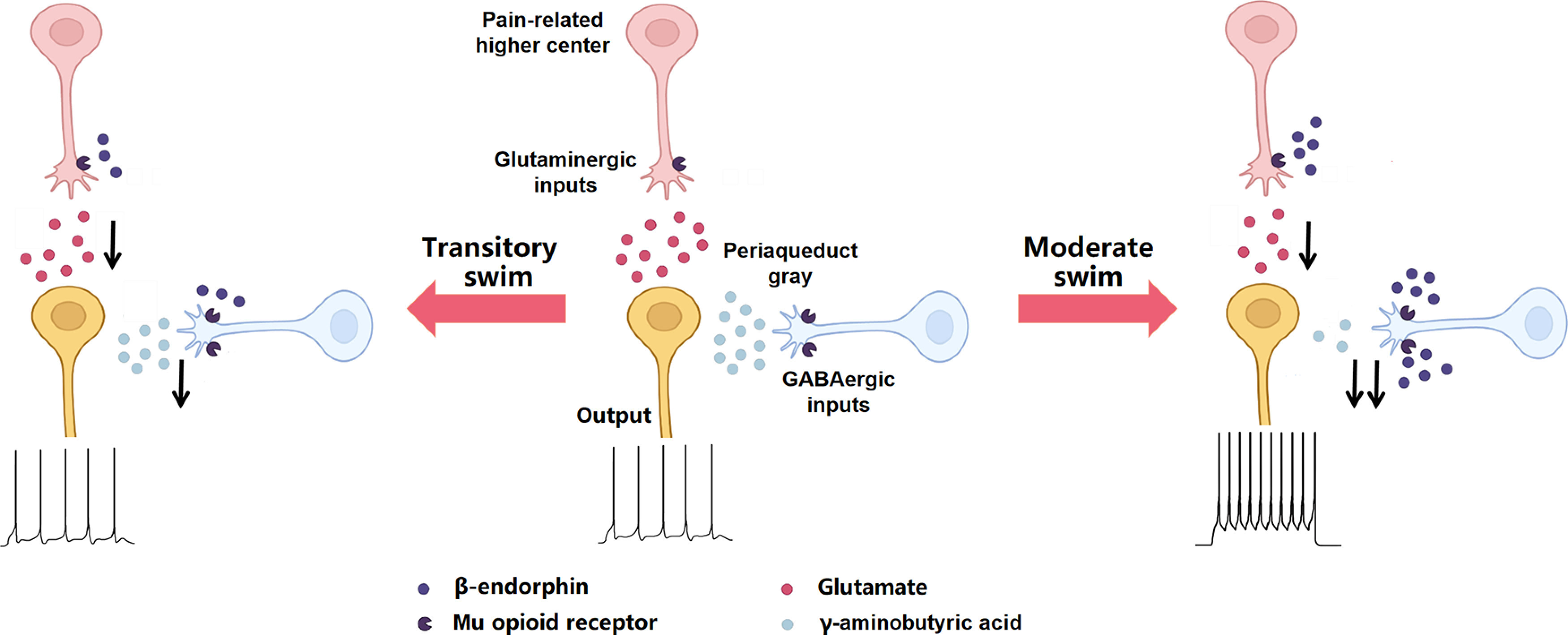
Schematic illustration showing the proposed mechanism of the contributions of MOR_GABA_ and MOR_Glut_ to analgesia induced by different intensities of stress. Under transitory swim stress, the activation of MOR_Glut_ and MOR_GABA_ by the released β-endorphin equally inhibits glutamatergic and GABAergic inputs on PAG-RVM neurons, respectively. These opposite effects on PAG-RVM neurons should neutralize each other and keeps the activity of PAG-projecting neurons almost unchanged. In contrast, under moderate swim stress, MOR_GABA_ are further activated by the more elevated β-endorphin to cause imbalance of excitatory–inhibitory synaptic inputs on PAG neurons, and therefore leads to disinhibition of PAG-RVM neurons and induces analgesia.

Stress-induced analgesia is a pain-inhibitory reaction usually occurs during or following exposure to sudden environmental change in nature. Although the numbers of neurotransmitter and neuromedin are involved in this physiological process, the opioid-dependent mechanism is still considered as a critical constituent part of stress-induced analgesia (Konecka et al., 1985; Miczek et al., 1985; Menendez et al., 1993; Mogil et al., 1996; Larauche et al., 2012). Our present study used 32°C forced swim paradigm, a classic stress procedure to induce analgesia, in a range of time courses to investigate the contributions of MORs to stress-induced analgesia. After transitory swim exposure, a significant increase of β-EP levels (Fig. 1*D*) was detected without accompanying behavioral analgesia (Fig. 1*A*). Surprisingly, MOR_Glut_^−/−^ mice exhibited an obvious stress-induced analgesia, together with an enhancement of activities of PAG-RVM neurons responding to noxious stimulus. Thus, both MOR_Glut_ and MOR_GABA_ were endogenously activated by weak stress stimulus but played counteractive effects on analgesic modulation: activation of MOR_GABA_ tended to induce analgesia, whereas that of MOR_Glut_ was inclined to exert a anti-analgesic-like effect, and these two opposite effects on PAG-RVM neurons neutralized each other and no analgesic behaviors was observed (Fig. 7).

In contrast, a steadily stress-induced analgesia depending mainly on MORs was observed after moderate swim exposure, as supported by the evidence of further elevated β-EP (Fig. 1*D*) and the effect of MOR antagonist β-FNA (Fig. 1*B*). This MOR-dependent analgesia mainly relies on endogenous activation of MOR_GABA_ because selective deletion of MOR_GABA_ inhibited stress-induced analgesia in the same direction as did β-FNA administration. On the contrary, selective deletion of MOR_Glut_ did not affect stress-induced analgesia, indirectly showing an overwhelming electivity of MOR_GABA_ on stress-induced analgesia in this condition. With the functional enhancement of endogenous opioids in parallel with the increase in stress intensity, the effects of MOR_GABA_ hold a dominant position, even covering the influence of MOR_Glut_, on pain modulation. Thus, a prominently analgesic effect mainly elicited by MOR_GABA_ was highlighted in the behavioral test, and an enhancement of PAG-RVM neuron activation was observed. Therefore, our present study showed a shift of MOR effects from balance between excitatory glutamatergic neurons and inhibitory GABAergic neurons to biasing toward inhibitory GABAergic neurons, in accordance with stress intensity (Fig. 7). While prolonging the swim time, MORs no longer mediated stress-induced analgesia, suggesting that the endogenous opioid system lost the initiative of analgesia under heavy stress.

β-EP is involved in stress responses and is closely related to stress-induced analgesia. It is clear that the hypothalamic–pituitary–adrenal (HPA) axis is the first activated neural circuit in response to stress events, and β-EP secretion is fully mediated by activation of the HPA axis. The transcriptional and translational levels of β-EP are significantly increased when the release of corticotropin-releasing hormone induced by activation of the HPA axis (Tsigos and Chrousos, 2002). In addition, β-endorphinergic neurons in the CNS are extensively projected into a pain-mediated area including hippocampus, pituitarium, and brainstem, and the delivered β-EP is highly degradation resistant in the brain (Smyth, 2016). Therefore, β-EP can be immediately secreted and distributed throughout the brain and blood vessels at the early stages of stress events and exerts its biological function over a considerable period. However, the present study showed a conflicting result that a significant increase of β-EP levels in plasma did not follow with expected analgesia under transitory swim exposure (Fig. 1*A*,*D*). In fact, the mismatch between the expression of opioid-dependent analgesia and enhancement levels of β-EP occurs frequently in several stress paradigms (Scallet, 1982; Izquierdo et al., 1984; Konecka et al., 1985; Külling et al., 1988). Just 24 h of food deprivation could significantly promote concentrations of β-EP twice the basal level in mice blood plasma, whereas significant analgesia needs food deprivation for 48 h without a further change in β-EP concentration (Konecka et al., 1985). In addition, MOR-dependent analgesia induced by social conflict needs aggressive confrontation between two populations of mice, but mere exposure of a test mouse to a nonaggressive opponent also provokes the changing of β-EP levels in pain-related brain areas (Külling et al., 1988). A similar phenomenon can be found in aversive sound and conditioned fear stress. The analgesia exhibited is hysteresis by the time β-EP levels have already elevated (Scallet, 1982; Izquierdo et al., 1984). Our results provide a potential explanation from another perspective for the mismatch between analgesia and β-EP: the neutralizing effects of MOR_Glut_ and MOR_GABA_, although further investigations are required for the distinct mechanisms.

The various expressions of MORs in glutamatergic and GABAergic neurons have clearly been identified recently, and both of them are oppositely involved in pain modulation with distinct mechanisms (Zhang et al., 2020). It was suggested that the exogenous opioid-induced analgesia is mediated by MOR_Glut_, whereas the endogenous opioid-induced analgesia is mediated by MOR_GABA_ (Zhang et al., 2020). Our present study genetically manipulated MORs expressed in glutamatergic or GABAergic neurons and found that, although transitory swim exposure failed to trigger remarkable analgesia in wild-type and MOR_GABA_^−/−^ mice, MOR_Glut_^−/−^ mice exhibited significant analgesic effect after stress stimulus. Previous studies about pain modulation of glutamatergic neuron MORs have mainly concentrated on exogenous opioid drug-induced pain modulatory effects (Kemp et al., 1996; Wang et al., 2018; Zhang et al., 2020). Based on the morphologic evidence that *vGlut2*-positive rather than *vGlut1*-positive neurons are densely distributed on brainstem and spinal cord (Soiza-Reilly and Commons, 2011), it has been verified that MORs expressed in *vGlut2*-positive neurons strongly inhibit pain signal transduction at the spinal level, but barely have effects on endogenous opioid-induced analgesia (Zhang et al., 2020). However, MORs are also extensively expressed in *vGlut1*-positive neurons at pain-related higher centers like primary and secondary somatosensory cortex, ACC, and hippocampus (Shi et al., 2020; Zhang et al., 2020), and they project to downstream pain-related areas of brainstem like PAG (Basbaum and Fields, 1984). Our present study indicated that MORs in *vGlut1*-positive neurons are also involved in endogenous opioid-induced pain modulation and, other than exogenous opioid-dependent analgesic effects at spinal level, they exhibit a novel anti-analgesic-like effects under the transitory forced swim paradigm. These results suggest a cell subtype specificity of glutamatergic neuron MORs on pain modulation.

By contrast, moderate forced swim paradigm-induced analgesia was significantly decreased in MOR_GABA_^−/−^ mice but was impervious in MOR_Glut_^−/−^ mice, indicating that the main mediation of analgesia is accomplished by GABAergic neuron MORs under this condition. This is consistent with the GABA disinhibition hypothesis of stress-induced analgesia (Yeung et al., 1977; Behbehani and Fields, 1979; Basbaum and Fields, 1984). Activation of MORs expressed on GABAergic neurons are suggested to attenuate their inhibition of the descending pain inhibitory pathway in brainstem or excitatory neurons projecting to the descending pain inhibitory pathway at prefrontal cortex and hippocampus, so that analgesia was induced (Lau and Vaughan, 2014).

In the CNS, modulation of stress-induced analgesia is mainly accomplished by the activation of the descending pain inhibitory pathway (Bourne et al., 2014; Kwon et al., 2014; Huang et al., 2019; Holgersen et al., 2021). The descending pain inhibitory pathway consists of PAG, RVM, and spinal dorsal horn. Activation of this circuit abolishes nociceptive transmission from dorsal horn of the spinal cord to supraspinal pain-related brain areas, resulting in inhibition of nociceptive signaling and antinociceptive effects (Bourne et al., 2014; Kwon et al., 2014; Ferdousi and Finn, 2018; Huang et al., 2019). As the origin of the descending pain pathway, neurons projecting from PAG to RVM play a vital role in the collection of nociceptive information from forebrain and the mediation of endogenous analgesia (Heinricher, 2016; Chen and Heinricher, 2019). MORs are generally located at the axons, terminals, dendrites, and somata of neurons (Drake and Milner, 2002) to depress the firing rate and neurotransmitter release of neurons by a G-protein-mediated inhibition process (Drake and Milner, 2002). Thus, endogenous activation of MOR_Glut_ inhibits the pain-related excitatory inputs to the descending pain pathway, whereas the activation of MOR_GABA_ disinhibits this circuit. Based on the extensive distributions of both GABAergic and glutamatergic neuron MORs on the descending pain pathway and pain-related higher brain areas projecting to this circuit, respectively, we evaluated the changes of PAG-RVM neuron activities by detecting calcium signals under transitory or moderate swim exposure to further investigate cell type-based differential MORs-mediated stress-induced analgesia. The enhancement of calcium activities on nociceptive neurons under thermal or mechanical noxious stimulus has been reported in several studies (Samineni et al., 2019; Huang et al., 2020; Moriya et al., 2020; Zhang et al., 2020), and efficient involvement of these projection neurons in the mediation of thermal noxious stimulus was verified in the present study.

Under transitory swim exposure, the calcium activities of PAG-RVM neurons poststress were unchanged in wild-type mice, but were significantly increased in MOR_Glut_^−/−^ mice and obviously decreased in MOR_GABA_^−/−^ mice (Fig. 5), suggesting that both MOR_Glut_ and MOR_GABA_ are involved in transitory swim exposure stress and exhibit an oppositely mediated effect in the descending pain inhibitory pathway. The activation of MORs expressed in GABAergic neurons elicits “disinhibition” of PAG-RVM neurons to induce analgesia, as it is uncovered by MOR_Glut_ deletion (Fig. 6*C*,*F*). In contrast, the activation of MORs expressed in glutamatergic neurons inhibits PAG-RVM neurons to cause antianalgesia, as it is uncovered by MOR_GABA_ deletion (Fig. 6*B*,*E*). Taking one with another, the opposite effects of MORs on the activities of PAG-RVM neurons through these two cell-type neurons might be neutralized (Fig. 7), so we did not detect a significant change of both tail-flick test and calcium activities in wild-type mice. This might be one reason to explain the inconsistency between elevated β-EP levels and missed analgesia in the behavioral test.

By contrast, under moderate swim exposure, the activities of PAG-RVM neurons in both wild-type and MOR_Glut_^−/−^ mice exhibited significant enhancements, whereas those in MOR_GABA_^−/−^ mice showed a lack of this enhancement, revealing a prominent effect of MOR_GABA_-mediated GABA disinhibition in the descending analgesic pathway on forced swim stress-induced analgesia (Lau and Vaughan, 2014). Under this stress condition, the inhibitory effect of MOR_Glut_ on PAG-RVM neurons was remarkably overwhelmed by the disinhibitory effect of MOR_GABA_; therefore, the descending pain inhibitory pathway was activated, and behavioral analgesia was detected (Fig. 7). Although electrophysiological evidence is still lacking to support that GABA disinhibition mechanism in acute stress is the major source of MOR-dependent analgesia, our results provide a new perspective to investigate the role of GABAergic neuron MORs on pain modulation pathways at cellular levels. However, the distinct mechanisms underlying the shifted weight of MOR_Glut_ and MOR_GABA_ efficiencies on the descending pain inhibitory pathway in response to different stress intensities remain speculative. It seems that the amount of β-EP related to stress intensity and the asymmetric distribution of MORs on glutamatergic and GABAergic neurons in the descending inhibitory pathway might serve as a key parameters to modulate the descending inhibitory pathway.

Strangely, transitory swim exposure did not induce obvious analgesia in MOR_GABA_^−/−^ mice, but calcium activities of PAG-RVM neurons was significantly weakened. This inconsistency between behavioral performance and the activities of PAG-RVM neurons might be attributed to the character of the descending pain inhibitory pathway, because it is a pain information modulatory circuit rather than a pain information transmittal circuit (Bourne et al., 2014; Kwon et al., 2014; Huang et al., 2019). Previous pharmacological research has already clarified that lidocaine, a sodium-channel blocker untouched by MORs, attenuated the chronic allodynia of mice through local administration into PAG and RVM, whereas they have no effect on tail-flick latency in healthy mice with the same treatment (Pertovaara et al., 1996). So, inhibition of the descending pain inhibitory pathway will not produce a direct hyperalgesic effect.

Overall, our present study highlighted the roles of MORs expressed in different populations of cells on MOR-dependent analgesia underwent various intensities of stress. However, the distinct mechanisms underlying the shifts of MOR_Glut_ and MOR_GABA_ efficiencies responding to stress intensity remain to be further explored. In addition, given that acute stress induces sex differences of analgesia in both laboratory animals and humans (Mogil, 2012), our present study of males should not generalize to females. It is worth investigating whether the sex-specific modulation of MORs in pain processing is involved in the present study.

## Synthesis

Reviewing Editor: Laura Bradfield, University of Technology Sydney Decisions are customarily a result of the Reviewing Editor and the peer reviewers coming together and discussing their recommendations until a consensus is reached. When revisions are invited, a fact-based synthesis statement explaining their decision and outlining what is needed to prepare a revision will be listed below. The following reviewer(s) agreed to reveal their identity: NONE.

Thank you for so thoroughly addressing the majority of the comments in the reviews. Almost everything has been satisfactorily addressed. There are two minor points remaining:

1. I previously requested that the significance statement be amended to include more information about the potential real-world significance of the findings, and this does not seem to have occurred. I wonder, is it possible that differential stress levels might affect the efficacy of opioid-based pharmacotherapies, for example? Or could it even contribute to substance use disorder development if, say, larger quantities of the drug are necessary for an effect in stress-affected individuals? If the author’s feel that such implications are beyond the scope of their work then it is not necessary to include them, but if they do I think it could increase the broadness of the appeal of the findings.

2. Reviewer #2 has requested the following: “The authors have fully addressed all of my concerns and the revised manuscript is suitable for publication. One minor suggestion is to reverse the order of Figure 1 and Figure 2 such that they are presented in the order they are discussed in the text of the results section (B-EP and MORs effects on stress-induced analgesia behavior, then RNAscope data in conditional knockout mice).” Please make sure this is addressed.

Finally, I would like to note that there are some areas where the grammar/phrasing is not ideal. If the authors could please check over this carefully it will improve readability.
